# Pattern of Endodontic Lesions of Maxillary and Mandibular Posterior Teeth: A Cone-Beam Computed Tomography Study

**DOI:** 10.3390/jimaging8100290

**Published:** 2022-10-20

**Authors:** Neda Hajihassani, Masoumeh Ramezani, Maryam Tofangchiha, Fatemeh Bayereh, Mehdi Ranjbaran, Alessio Zanza, Rodolfo Reda, Luca Testarelli

**Affiliations:** 1 Department of Endodontics, Dental Caries Prevention Research Center, Qazvin University of Medical Sciences, Qazvin 3419915315, Iran; 2 Department of Oral and Maxillofacial Radiology, Dental Caries Prevention Research Center, Qazvin University of Medical Sciences, Qazvin 3419915315, Iran; 3 Dental Caries Prevention Research Center, Qazvin University of Medical Sciences, Qazvin 3419915315, Iran; 4 Metabolic Diseases Research Center, Research Institute for Prevention of Non-Communicable Diseases, Qazvin University of Medical Sciences, Qazvin 3419915315, Iran; 5 Department of Oral and Maxillofacial Sciences, Sapienza University of Rome, 00161 Rome, Italy

**Keywords:** cone-beam computed tomography, maxilla, mandible, periapical lesions, root canal therapy

## Abstract

The pattern of expansion of endodontic lesions in the jaws has been less commonly addressed in the literature. For this reason, the aim of this study is to assess the pattern of endodontic lesions of maxillary and mandibular posterior teeth using cone-beam computed tomography (CBCT). This cross-sectional study was conducted on 317 endodontic lesions of posterior teeth on CBCT scans retrieved from a radiology center in Qazvin, Iran, from 2020 to 2022. Endodontic lesions were assessed on sagittal, coronal, and axial sections by an endodontist and dental student using the Romexis software. The largest lesion diameter was measured occluso-apically, mesiodistally, and buccolingually. Lesion size was analyzed based on age, gender, jaw, tooth type, and presence/absence of root filling by independent samples *t*-tests and a one-way Analysis Of Variannce (ANOVA). The largest diameter of lesions in the maxilla and mandible was recorded in the occluso-apical dimension followed by buccolingual and mesiodistal dimensions (*p* > 0.05). The pattern of lesions was the same in teeth with and without endodontic treatment, but it was significantly different in maxillary and mandibular endodontically treated teeth in the occluso-apical and buccolingual dimensions (*p* < 0.05). No significant correlation was noted with tooth type or jaw except for maxillary and mandibular first molar lesions, which were significantly different in the occluso-apical dimension (*p* < 0.05). Lesion size in all three dimensions was significantly greater in males than females (*p* < 0.05), and was the highest in the occluso-apical dimension in both genders. In the maxilla, the mean lesion size significantly decreased in the mesiodistal dimension with age (*p* < 0.05). In conclusion, the largest lesion diameter in the maxilla and mandible was found in the occluso-apical dimension, indicating the role of bone density in the pattern of lesions.

## 1. Introduction

Radiolucent lesions of the jaws include a wide range of odontogenic and non-odontogenic lesions [1,2]; among which, inflammatory lesions are the most common and often appear in the form of a radiolucency related to a necrotic tooth [3]. The presence of microorganisms in carious teeth or exposure of pulp tissue to oral microorganisms are the main causes of pulpitis and the development of periapical (PA) lesions. Persistent irritation by the microorganisms results in widespread inflammation in the pulp tissue, which can lead to pulpal necrosis [4]. Next, the microorganisms invade the apical and PA region through the root canal and cause an inflammatory PA lesion [5,6,7].

Evidence shows that a bone lesion can be detected on bidimensional radiographs when 30% to 50% of bone mineral content is lost, or when the lesion extends to the interface of cortical and cancellous bones [5]. Despite the fact that PA radiography is the standard reference for the radiographic diagnosis of PA lesions [8], its diagnostic accuracy depends on several factors [5], such as the size and extension of the lesion in bone [9,10], the internal structure of the lesion [10], the image contrast [5], X-ray irradiation angle [11], tooth position, and the surrounding bone density [5].

PA lesions smaller than 1–2 mm are not detectable on PA radiographs [5], which can cause problems in the primary detection of root involvement, especially when clinical signs and symptoms indicate pulp necrosis or irreversible pulpitis [8].

It has been well documented that cone-beam computed tomography (CBCT) is much more accurate than PA radiography for the detection of PA lesions due to its high resolution and accuracy in the 3D evaluation of teeth and their surrounding structures [8,12]. In CBCT, data are reconstructed based on CT-based algorithms in order to provide 3D images in sagittal, coronal, and axial planes; such details cannot be detected in conventional 2D images [8,13].

Several studies have compared the accuracy of CBCT with panoramic and PA radiographic modalities for the detection of PA lesions and have reported a higher accuracy in CBCT than in 2D modalities [5,12,13,14,15,16,17]; therefore, the application of CBCT for the diagnosis or management of endodontic problems is on the rise [8,18,19,20]. Moreover, CBCT, when prescribed for the right reason [21], provides important anatomical information for the management of root canal treatment and visualization of the root canal anatomy, leading to decreased rate of treatment failure [22,23,24].

The pattern of expansion of endodontic lesions in the jaws has been less commonly addressed in the literature [5]. Considering the significance of the correct and early detection of lesions for proper treatment planning, for orthograde endodontic therapy or radicular surgery [14,18], plus the higher accuracy of CBCT for diagnostic purposes, especially for detection of buccolingual lesions compared with 2D radiography [13], this study aims to assess the pattern of endodontic lesions of posterior maxillary and mandibular teeth using CBCT.

## 2. Materials and Methods

This cross-sectional study was conducted on 317 CBCT scans of patients retrieved from the archives of an oral and maxillofacial radiology clinic in Qazvin city, Iran, during 2020–2022. The study was approved by the ethics committee of the respective university (IR.QUMS.REC.1401.060).

### 2.1. Eligibility Criteria

The inclusion criteria were: (I) optimal-quality radiographs (no motion artifacts and the optimal resolution of images), (II) having at least one posterior maxillary or mandibular tooth with an endodontic lesion, and (III) the availability of demographic information of patients such as age and gender.

The exclusion criteria were open-apex teeth, visible root fracture, internal resorption, external resorption not related to root apex, lesions with non-differentiable borders, and pathological lesions with an origin other than the pulp and peri-apex.

### 2.2. Sample Size Calculation

The sample size was calculated to be 129 assuming the maximum standard deviation of lesion length to be 1.9 mm [5], a type one error of 0.05, and a maximum estimated error of 0.33.

### 2.3. Image Assessment

**Figure 1:**
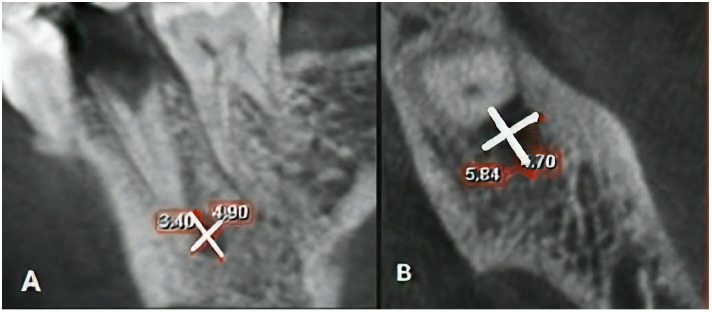
Measuring the dimensions of an endodontic lesion: (**A**) measuring the largest dimension of lesion in occluso-apical and mesio-distal dimensions on the sagittal view; (**B**) axial view.

All images were obtained with a ProMax 3D CBCT scanner (Planmeca, Helsinki, Finland) with 0.15 × 0.15 mm voxel size and 8 × 8 cm field of view. First, the lesion’s borders were identified and marked by an endodontist and a trained senior dental student with a 95% inter-examiner agreement. In cases where the two observers did not agree on a lesion border’s location, an experienced oral and maxillofacial radiologist was requested to help them reach a consensus. Next, the parameters were measured by a trained senior dental student. All images were assessed in a semi-dark room on a 19-inch monitor (Samsung, Seoul, Korea) by the Romexis version 3.8.3 software (Planmeca, Helsinki, Finland), and the sagittal, coronal, and axial sections were assessed. The largest diameter of lesions in the occluso-apical, mesio-distal, and bucco-lingual dimensions was measured in millimeters (mm) and recorded along the longitudinal axis of the involved tooth (Figure 1).

To determine the location of lesions, the tooth root was measured from the cementoenamel junction to the apex and hypothetically divided into three equal parts, cervical, middle, and apical. Apical lesions were considered as those in the apical third, lateral lesions were those in the middle third, latero-apical lesions were those located in the middle third to apical third, furcal lesions were those located in the furcation area, and furcal-apical lesions were those located between the furcation area and apical lesion.

### 2.4. Statistical Analysis

Data were analyzed by SPSS version 25 (IBM Co., Armonk, NY, USA). The frequency and percentage values were reported for qualitative variables, and the mean and standard deviation values were reported for quantitative variables. The normality of the dependent variables was analyzed and confirmed through a histogram and normal QQ plot. Independent samples *t*-tests, Pearson’s correlation, and a one-way ANOVA were used for general comparisons; in addition, post-hoc tests were applied for pairwise comparisons. The level of significance was set at 0.05.

## 3. Results

A total of 317 lesions were evaluated, out of which, 174 lesions were in females (54.9%) and 143 lesions were in males (45.1%). Additionally, 72 lesions (22.7%) were related to teeth without endodontic treatment, and 245 (77.3%) were related to endodontically treated teeth. Of all, 181 lesions (57.1%) were in the mandible and 136 (42.9%) were in the maxilla. Table 1 shows the location of the lesions and the types of roots with endodontic lesions.

The mean age of patients was 41.82 ± 12.24 years (ranged 14 to 73 years). In total, the mean mesiodistal, buccolingual, and occluso-apical dimensions of lesions were 3.73 ± 2.05 mm (ranged 0.86 to 12.09 mm), 3.82 ± 1.82 mm (ranged 0.80 to 12.42 mm), and 4.22 ± 2.69 mm (ranged 0.62 to 16.65 mm), respectively. Table 2 compares the mean dimensions of lesions in males and females. According to the *t*-tests, the mean size of the lesions in all three dimensions was significantly larger in males than in females (*p* < 0.05).

The highest frequency of lesions in both jaws was at the apex of the teeth. Moreover, of the roots involved with a lesion, the mesio-buccal root of maxillary molars and the mesial root of mandibular molars had the highest frequency. Among the maxillary premolars, eight teeth had two roots; of which, five teeth had buccal, and three had palatal lesions; thus, in total, buccal roots had a higher frequency of lesions than palatal roots in the maxilla.

According to the *t*-tests, the mean size of endodontic lesions was not significantly different in teeth with and without endodontic treatment in mesiodistal (*p* = 0.424), buccolingual (*p* = 0.899), and occluso-apical (*p* = 0.574) dimensions, irrespective of the type of jaw, gender, and other variables.

Table 3 presents the mean dimensions of endodontic lesions in the maxilla and mandible based on the presence and absence of endodontic treatment. As shown, in the maxilla, the lesions in an absence of endodontic treatment were larger than those in teeth with endodontic treatment in all three dimensions, and this difference was significant in the buccolingual (*p* = 0.05) and occluso-apical (*p* = 0.034) dimensions. In the mandible, this difference was not significant in any dimension (*p* > 0.05).

In a comparison of teeth in the maxilla and mandible, the *t*-tests showed that this part of the maxillary lesions without endodontic treatment was larger than the mandibular lesions in all three dimensions but not significantly (*p* > 0.05). In endodontically treated teeth, mandibular lesions were larger than the maxillary lesions in all three dimensions; however, this difference was only significant in the occluso-apical (*p* = 0.002) and buccolingual (*p* = 0.001) dimensions. In both the maxilla and mandible, lesions in endodontically treated teeth had the largest size in the occluso-apical dimension (*p* < 0.05, Table 4). 

Regarding the correlation of lesion size with age, the results showed a significant inverse correlation between age and lesion size in mesiodistal dimensions (−0.194, *p* = 0.024) in the maxilla; in other words, by an increase in age, the size of the lesion in the mesiodistal dimension significantly decreased. No other significant correlation was found in the maxilla or mandible (*p* > 0.05).

Table 5 presents the mean size of lesions in the three dimensions based on tooth type. The one-way ANOVA showed no significant correlation between lesion size and type of tooth, neither in the maxilla nor in the mandible (*p* > 0.05). In addition, the *t*-tests found no significant difference between the lesion size of mandibular teeth in comparison with their corresponding maxillary teeth (*p* > 0.05). However, the occluso-apical dimension of lesions of mandibular first molars was significantly larger than that of maxillary first molars (*p* = 0.045).

The smallest lesion size in the mesiodistal dimension was recorded in furcal lesions of maxillary teeth, while the largest lesion size in the occluso-apical dimension was recorded in furcal to apical lesions of mandibular teeth; however, no significant correlation was found between lesion location and size of the lesion, neither in the maxilla nor in the mandible (*p* > 0.05).

According to the *t*-tests, no significant correlation existed between the lesion size in any dimension and its buccal or palatal location in the maxilla (*p* > 0.05).

## 4. Discussion

This study assessed the pattern of endodontic lesions in the posterior maxilla and mandible using CBCT. The results showed that the pattern of endodontic lesions was not significantly different in teeth with and without endodontic treatment. In the maxilla, the size of lesions related to teeth with no endodontic treatment, in the bucco-lingual and occluso-apical dimensions, was significantly larger than that in lesions of endodontically treated teeth. This finding was expected since the source of infection, necrotic tissue, and microorganisms are still present in teeth without endodontic treatment [4,7,25]; thus, the lesion size and speed of lesion expansion are expected to be higher in teeth with active infection and no endodontic treatment. The present results found no significant correlation between the lesion size and endodontic treatment of the respective teeth in the mandible.

Comparison of the pattern of lesions in maxillary and mandibular teeth without endodontic treatment revealed no significant difference, which may be due to the small number of lesions in non-endodontically treated teeth, as one indication of a CBCT request in endodontics is the assessment of the causes of endodontic treatment failure, and evaluation of the size and healing status of PA lesions after endodontic treatment [17,18]. For teeth with pulpitis or necrosis (due to severe caries or other reasons) that have not yet undergone endodontic treatment, diagnosis and treatment planning are often performed based on panoramic and PA radiographs [15], and CBCT is requested only for certain cases. Therefore, the number of available CBCT images for the assessment of endodontic lesions of non-endodontically treated teeth was highly limited.

Comparison of the lesion size in endodontically treated maxillary and mandibular teeth showed significantly larger lesions in the occluso-apical and bucco-lingual dimensions in endodontically treated mandibular teeth. Considering the density of buccal and lingual cortical bones in the mandible and their resistance against horizontal infection spread, lesions further expand in size vertically rather than horizontally.

Despite the effect of different factors on the pattern of endodontic lesions in the jaws, most studies have assessed the prevalence of endodontic lesions [2,3,5,6,9,26,27], and studies on the effect of gender on the pattern of endodontic lesions are limited [3,5,27]. The present results revealed a significantly larger size of lesions in all three dimensions in males. In addition, in both males and females, lesions had the highest extension in the occluso-apical dimension. Since females pay more attention to their dental health than males, the possibility of sooner detection of endodontic lesions on routine dental X-rays is higher in females, and thus, lesion size is smaller in them. Similarly, Kazemipoor and Sabaghzadegan [5] indicated that the expansion of PA lesions in anterior teeth in all three dimensions was significantly greater in males than females, and the maximum mean size of lesions was recorded in the vertical dimension in males, and the buccolingual horizontal dimension in females.

Assessment of the correlation of age with the size of endodontic lesions in the present study showed that the mean size of lesions in the mandible increased with age; however, this association was not significant. In general, bone resorption increases, and bone density decreases with age; thus, with an increase in age, bone destruction by endodontic inflammatory lesions increases [5]. Additionally, the present results indicated that in the maxilla, lesion size decreased with age; however, this correlation was only significant in the mesiodistal dimension. Smaller lesion size in the maxilla can be due to the fact that the dimensions of the maxillary sinuses increase in bone with age; thus, the available bone in the maxilla decreases. Resultantly, older individuals have smaller lesions than younger individuals due to a lower amount of available bone for the enlargement of inflammatory lesions. Kazemipoor and Sabaghzadegan [5] evaluated the size of endodontic lesions in three age groups and found significant differences in the size of lesions in vertical and mesiodistal dimensions in 15–44 year-olds, and the mesiodistal and buccolingual dimensions in 45–54 year-olds; however, no difference was found in 55–79-year-olds. Despite the differences in the structure of maxillary and mandibular bone, the effect of age on the pattern of lesions was not separately evaluated in each jaw in their study.

No significant correlation was noted between lesion size and location in the present study; however, the majority of lesions were apical lesions in both jaws, which was expected, since the path of odontogenic infections is through the main canal to the apex [25,28]. In addition, a study of the prevalence of accessory canals in different parts of the teeth revealed that 74% of accessory canals are in the apical third, 15% are in the cervical third, and 11% are in the middle third; thus, higher frequency of apical lesions is justified [29,30]. Meng et al. [10] evaluated the correlation of roots and endodontic lesions in the sagittal plane and reported that root and radicular cysts mostly had a centripetal relationship, and only a small number of cysts had horizontal expansion towards the palatal or buccal surface.

In the present study, the frequency of furcal and furcal to apical lesions was significantly higher in the mandible than maxilla, which was expected considering the root anatomy of mandibular and maxillary molars, since the roots are more divergent in the mandible and dentin thickness is thinner at the furcation site. Moreover, several accessory foramina are present in the furcation area of mandibular molars, which cannot be cleaned and shaped [30]; thus, the risk of developing furcal lesions is higher in the mandible.

In the maxilla, buccal roots had a higher rate of involvement than palatal roots, and the mean size of buccal lesions in all three dimensions was larger than palatal lesions, but not significantly. Since buccal roots are shorter and thinner than palatal roots [31], inflammation is faster transferred along the canal to the apex [28]; moreover, the risk of procedural errors is higher in buccal roots due to more difficult access to the canal orifice, greater root curvature, thinner roots, and a higher possibility of additional and accessory canals in buccal roots compared with palatal roots. Furthermore, in 30% to 50% of the cases, an isthmus exists at the conjunction of middle and apical thirds in the mesiobuccal roots of maxillary first molars, which may be missed in the process of cleaning and shaping [29].

In the mandible, mesial roots showed higher involvement than distal roots in the present study. Since mesial roots have greater curvature than distal roots [29], they are at higher risk of procedural errors such as strip perforation. Further, it has been demonstrated that 80% of mesial roots of mandibular first molars have an isthmus in the apical to the middle third of the root, which contains pulp tissue, and the cleaning and shaping of this area are highly difficult if not impossible; thus, it may remain and serve as a source of infection [29,30].

In the present study, the mean size of lesions in the posterior teeth was the largest in the occluso-apical, followed by the buccolingual and then mesiodistal dimensions. In the study by Kazemipoor and Sabaghzadegan [5], the highest mean size of lesions in the anterior teeth was reported in the occluso-apical followed by the buccolingual and mesiodistal dimensions. This pattern may be explained by the path of odontogenic infections in the canal from the cervical towards the apical, which results in the vertical extension of lesions [7]. Lesion size is limited horizontally by the cortical bone. Considering the difference in thickness of cortical and cancellous bones in the posterior maxilla and mandible [27], this limitation is more pronounced in the mandible. Therefore, the lesion would expand in a vertical dimension. The present results and those of Kazemipoor and Sabaghzadegan [5] have indicated that the pattern of endodontic lesions can be the same in posterior and anterior teeth; however, further studies on different populations are still required on this topic. 

The frequency and mean size of lesions in all three dimensions were greater in the mandible than in the maxilla; this finding was expected considering the fact that the majority of CBCT scans evaluated in the present study had been requested for assessment of the position of mandibular third molars relative to the mandibular canal, height, and thickness of bone for implant placement, or distance of the implant from the critical structures such as the mandibular canal and mental foramen, and endodontic lesions were accidental findings on CBCT scans. Although this difference was not significant in the present study, further studies with a larger sample size may find significant differences in this regard.

The frequency of endodontic lesions in the molar teeth of both jaws was higher than in premolars in the present study. Additionally, mandibular first molars had the highest frequency, and mandibular first premolars had the lowest frequency of endodontic lesions. No significant difference was noted between the pattern of lesions in mandibular posterior teeth compared with the corresponding teeth in the maxilla. However, this difference was significant in the occluso-apical dimension of maxillary and mandibular first molars. Mandibular and maxillary molars are among the first permanent teeth to erupt at the age of 6–7 years, and thus, they are at higher risk of caries [29].

Single-center design and using CBCT scans available in the archives of one radiology center were among the limitations of this study, which limit the generalization of results to other populations. Since no information was available regarding the clinical examination of patients, the exact cause of endodontic lesions was not known. In addition, the number of images of teeth with PA lesions without endodontic treatment was low since such cases are often detected on PA and panoramic radiographs, and CBCT is requested only when vertical root fracture is suspected.

Future studies with a larger sample size on different populations are required to obtain more reliable results. Additionally, similar studies are recommended to find the pattern of endodontic lesions in endodontically treated teeth based on the type of procedural error.

## 5. Conclusions

The largest lesion diameter in both the maxilla and mandible was found in the occluso-apical dimension followed by the buccolingual and mesiodistal dimensions, indicating the role of bone density in the pattern of lesions. The lesions were larger in non-endodontically treated maxillary teeth, endodontically treated mandibular teeth, in males, at the mesial roots of mandibular teeth, and at the mesiobuccal root of maxillary teeth. The highest frequency of lesions in both jaws was at the apical area. Moreover, age had an inverse correlation with lesion size in the mesiodistal dimension.

## Figures and Tables

**Table 1:** Location of lesions and type of root with endodontic lesion.

Title	Location/Jaw	Jaw/Location	Number	Percentage
Location of lesions	Apical	Maxilla	102	75.00
		Mandible	121	66.90
	Lateral	Maxilla	2	1.50
		Mandible	4	2.20
	Latero-apical	Maxilla	23	16.90
		Mandible	27	14.90
	Furcal	Maxilla	6	4.40
		Mandible	18	9.90
	Furcal to apical	Maxilla	3	2.20
		Mandible	11	6.10
Jaw	Maxilla	Palatal	26	19.10
		Buccal (in premolars)	8	5.90
		Mesiobuccal	48	35.30
		Distobuccal	15	11.00
	Mandible	Mesial	75	41.40
		Distal	66	36.50

**Table 2:** Comparison of the mean (±standard deviation) dimensions of lesions in males and females.

Gender		Mesiodistal	Buccolingual	Occluso-Apical
Females	Mean ± std. deviation	3.46 ± 1.80	3.65 ± 1.68	3.97 ± 2.26
Males	Mean ± std. deviation	4.06 ± 2.18	4.05 ± 1.95	4.52 ± 2.98
	*p* value	0.009	0.048	0.046

**Table 3:** Mean dimensions of endodontic lesions in the maxilla and mandible based on presence and absence of endodontic treatment.

Endodontic Treatment		Number (%)	Mesiodistal	Buccolingual	Occluso-Apical
Maxilla	With endodontic treatment	100 (73.53)	1.62 ± 3.42	1.43 ± 3.40	3.62 ± 1.69
	Without endodontic treatment	36 (26.47)	1.92 ± 3.92	2.05 ± 4.03	4.38 ± 2.14
*p* value			0.120	0.050	0.034
Mandible	With endodontic treatment	145 (80.12)	3.86 ± 2.90	4.11 ± 1.91	4.55 ± 2.96
	Without endodontic treatment	36 (19.88)	3.87 ± 2.47	3.68 ± 1.93	4.36 ± 3.39
*p* value			0.947	0.228	0.737

**Table 4:** Mean dimensions of endodontic lesions in treated and non-treated teeth based on the type of jaw.

Endodontic Treatment	Jaw		Number (%)	Mesiodistal	Buccolingual	Occluso-apical
With Endodontic treatment		Maxilla	100 (40.81)	3.42 ± 1.52	3.40 ± 1.43	3.62 ± 1.69
		Mandible	145 (59.18)	3.86 ± 2.09	4.11 ± 1.91	4.55 ± 2.96
*p* value				0.074	0.001	0.002
Without endodontic treatment		Maxilla	36 (50.0)	3.92 ± 1.92	4.03 ± 2.05	4.38 ± 2.14
		Mandible	36 (50.0)	3.87 ± 2.74	3.68 ± 1.93	4.36 ± 3.39
*p* value				0.936	0.462	0.983

**Table 5:** Mean size of lesions in the three dimensions based on tooth type.

Jaw	Tooth Type	Number (%)	Mesiodistal	Buccolingual	Occluso-Apical
Maxilla	First premolar	21 (15.40)	3.29 ± 1.61	3.33 ± 1.27	3.71 ± 1.64
	Second premolar	24 (17.60)	3.35 ± 1.55	3.63 ± 1.59	4.01 ± 1.80
	First molar	75 (55.10)	3.72 ± 1.61	3.61 ± 1.63	3.89 ± 1.82
	Second molar	16 (11.80)	3.39 ± 2.03	3.58 ± 2.20	3.36 ± 2.30
	Total	Mean	3.55 ± 1.64	3.57 ± 1.64	3.82 ± 1.84
		*p* value	0.604	0.909	0.703
Mandible	First premolar	6 (3.30)	3.93 ± 2.23	4.27 ± 1.73	4.97 ± 2.74
	Second premolar	16 (8.80)	3.85 ± 2.07	3.40 ± 1.73	3.98 ± 2.74
	First molar	123 (68.00)	3.88 ± 2.24	4.12 ± 1.96	4.61 ± 3.20
	Second molar	36 (19.90)	3.80 ± 2.36	3.94 ± 1.93	4.36 ± 3.06
	Total	Mean	3.86 ± 2.30	4.02 ± 1.91	4.52 ± 3.05
		*p* value	0.998	0.544	0.845

## Data Availability

Not applicable.
